# Molecular Selection, Modification and Development of Therapeutic Oligonucleotide Aptamers

**DOI:** 10.3390/ijms17030358

**Published:** 2016-03-11

**Authors:** Yuanyuan Yu, Chao Liang, Quanxia Lv, Defang Li, Xuegong Xu, Baoqin Liu, Aiping Lu, Ge Zhang

**Affiliations:** 1 Institute for Advancing Translational Medicine in Bone & Joint Diseases, School of Chinese Medicine, Hong Kong Baptist University, Hong Kong, China; yu.yy01@hotmail.com (Y.Y.); liangchao512@163.com (C.L.); lvquanxia@163.com (Q.L.); lidefang@163.com (D.L.); 2 Zhengzhou Hospital of Traditional Chinese Medicine, Zhengzhou 450007, China; xuxg1115@126.com

**Keywords:** oligonucleotide aptamers, monoclonal antibodies, diseases therapy, preclinical study, clinical evaluation

## Abstract

Monoclonal antibodies are the dominant agents used in inhibition of biological target molecules for disease therapeutics, but there are concerns of immunogenicity, production, cost and stability. Oligonucleotide aptamers have comparable affinity and specificity to targets with monoclonal antibodies whilst they have minimal immunogenicity, high production, low cost and high stability, thus are promising inhibitors to rival antibodies for disease therapy. In this review, we will compare the detailed advantages and disadvantages of antibodies and aptamers in therapeutic applications and summarize recent progress in aptamer selection and modification approaches. We will present therapeutic oligonucleotide aptamers in preclinical studies for skeletal diseases and further discuss oligonucleotide aptamers in different stages of clinical evaluation for various disease therapies including macular degeneration, cancer, inflammation and coagulation to highlight the bright commercial future and potential challenges of therapeutic oligonucleotide aptamers.

## 1. Introduction

Monoclonal antibodies have been the dominant agents in the biomedical field for detection and inhibition of target molecules in biomedical research since they were introduced in 1975 [1]. Highly sensitive antibody-based diagnostics and therapeutics have been aggressively applied in industries without any intellectual property restriction [2]. However, the main issues of monoclonal antibodies are the high immunogenicity, low production, high cost and low stability. Recently oligonucleotide aptamers have become the most promising agents to compete with antibodies not only in diagnostics but also in therapeutics.

Aptamers are short (20–70 bases) single stranded oligonucleotides (ssRNA/ssDNA) which bind to their targets through 3D conformational complementarities with high affinity and specificity. The term aptamer is derived from a Latin word “aptus” with the meaning of “to fix”, indicating the lock and key relationship of aptamers for their targets [3,4]. Aptamers can be tailored selectively against various targets including nucleotides, amino acids, proteins, small molecules, virus and live cells [5]; proteins are the major targets in aptamer research.

Aptamers can be selected through an *in vitro* process called Systematic Evolution of Ligands by EXponential enrichment (SELEX), which was first developed by three groups independently in 1990 [3,4,6]. Compared to monoclonal antibodies, aptamers possess similar affinity and specificity, but have minimal immunogenicity, high production, low cost and high stability, making them the most advanced reagents for detection and inhibition of target molecules beyond monoclonal antibodies. Until now, there have been over 900 aptamers developed against various targets for diagnostic and therapeutic purposes [7]. For therapeutic applications, aptamers have been developed against a broad spectrum of diseases, including AIDS, cancer, diabetes, skeletal diseases. There are 11 aptamers under different stages of clinical trials for treatment of macular degeneration, cancer, coagulation and inflammation. Pegaptanib, an aptamer against vascular endothelial growth factor (VEGF), the first therapeutic aptamer approved by the FDA for the treatment of wet age-related macular degeneration (wet AMD), has been successfully used in market [8,9,10,11]. It opens a wide window for the following development of more therapeutic oligonucleotide aptamers.

In this review, we will first explain the advantages and limitations of oligonucleotide aptamers from the aspects of immunogenicity, production, cost and stability, and then talk about recent progress in optimization of aptamer selection process and downstream aptamer modifications. We will summarize therapeutic oligonucleotide aptamers in preclinical studies for skeletal diseases and further discuss oligonucleotide aptamers in different stages of clinical evaluation for various disease therapies including macular degeneration, cancer, inflammation and coagulation, to highlight the bright commercial future and potential challenges of therapeutic oligonucleotide aptamers. At the end, we will discuss the potential targets for developing therapeutic oligonucleotide aptamers based on the known targets of approved monoclonal antibodies, which will provide a clear direction for development of therapeutic oligonucleotide aptamers.

## 2. Monoclonal Antibodies *versus* Oligonucleotide Aptamers

### 2.1. Advantages of Oligonucleotide Aptamers

Aptamers possess similar affinity and specificity as monoclonal antibodies, but have some important advantages over antibodies. It is difficult to develop monoclonal antibodies with no immunogenicity, but aptamers are not recognized by the immune system as foreign and do not stimulate a negative immune response because of the small size (around 30 kDa) [12]. On the other hand, special modifications such as substitution of C or G with 2′-*O*-methylribonucleotide could avoid stimulating immune response [13,14,15]. There is no aptamer with high immunogenicity reported till now. Pegaptanib, the first aptamer approved by FDA for treating wet AMD showed no immunogenicity in either preclinical evaluation in animals or clinical trials in patients. For production and cost, identification of antibodies starts in mice and requires screening a series of cells, which is rather laborious and expensive. Aptamers are identified *in vitro* so the selection conditions can be controlled and adjusted on demand, and nonphysiological buffers or nonphysiological temperatures could be used if necessary. Aptamers can be easily but accurately synthesized by chemical methods, so production of large quantities of aptamers is less expensive and less risky [16]. More importantly, there is no batch to batch variation in aptamer production. For stability, antibodies are proteins, which are very sensitive to temperature and would be denatured or degraded easily under wrong storage or transport conditions. So antibodies have limited shelf life and require a continuous cold chain during transportation to avoid denaturation [5]. Aptamers have an indefinite shelf life as they are temperature resistant and can tolerate transportation without any special requirements for cooling. This eliminates the need for a continuous cold chain in long-term storage or transportation [5]. The function of aptamers could be regenerated easily even if they are denatured, as the denaturation could be easily reversed. Thus, aptamers display distinct advantages over monoclonal antibodies in both diagnostic and therapeutic applications.

### 2.2. Limitations of Oligonucleotide Aptamers

There are also some barriers for aptamer identification and application. Aptamers can be degraded by nuclease in serum and have short half-lives and can be cleared rapidly in the circulation due to their small size. Therefore, downstream modifications are needed before use *in vivo*. Aptamer modifications are rather sequence dependent and have a high risk of failing as modifications may affect folding structures of aptamers and lead to loss of function. Aptamers identified from SELEX that have high specificity *in vitro* may fail to inhibit their targets *in vivo* as expected. The successful rate of effective aptamer identification by conventional *in vitro* aptamer selection methods is lower than 30% [17]. Optimization of selection strategies or conjugation of specific aptamers to an effective therapeutic payload such as microRNAs/siRNAs/small molecule compounds/monoclonal antibodies to form nanocomplex (aptamer guided target delivery) for desired therapeutic aim can help to overcome this barrier [18].

### 2.3. Aptamer-Antibody Conjugation

**Figure 1:**
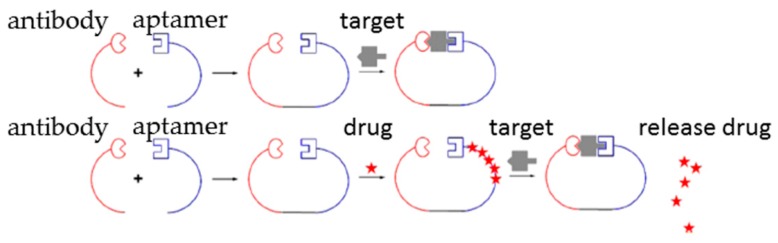
Aptamer-antibody conjugation can be used directly against the same target or for drug targeted delivery (Reproduced with permission from Reference [21]). **Upper**: Anti-thrombin antibody and anti-thrombin aptamer bind to different sites to thrombin. Conjugation of antibody and aptamer (AAP) has 100 and 35 fold higher affinity to thrombin than antibody and aptamer alone, respectively. For conjugation, amine-functionalized aptamer was maleimide activated by sulfosuccinimidyl 4-(*N*-maleimidomethyl) cyclohexane-1-carboxylate (sulfo-SMCC), and thio-functionalized antibody was conjugated to *N*-succinimidyl-S-acetylthioacetate (SATA). Then aptamer and antibody were mixed and incubated for covalent conjugation. **Lower**: Anti-human epidermal growth factor receptor 2 (HER2) aptamer was conjugated with anti-HER2 antibody by same conjugation method (AAP) and then loaded with doxorubicin (AAP-Dox). Folding of the aptamer which loaded Dox changes when aptamer binds to HER2, then Dox will be released from AAP-Dox. AAP-Dox has approximately three- and six-fold higher cytotoxicity than Dox alone and antibody alone, respectively [21].

Combination use of aptamers and antibodies is a novel therapeutic strategy that shows higher potency than using aptamers or antibodies individually in some cases [18,19,20]. Antibody-aptamer pincers (AAPs) have been developed to increase binding affinity and inhibition potency of antibodies or aptamers to their targets (Figure 1). Anti-thrombin aptamer and antibody conjugation that binds to different epitopes of thrombin have been designed. The AAP has significant lower dissociation constant value (*K_d_* = 567 pM) than the antibody alone (*K_d_* = 50 nM) or aptamer alone (*K_d_* = 3.5 nM) to thrombin [21]. In a breast cancer study, the AAP system was combined with human epidermal growth factor receptor 2 (HER2) drug targeted delivery system. Anti-HER2 aptamer loaded with doxorubicin is conjugated with anti-HER2 antibody to form an AAP-HER2-Dox drug targeted delivery system. This system has much higher cytotoxicity (*IC_50_* = 15.5 nM) to tumors than drug only (*IC_50_* = 43.9 nM) or aptamer loaded with Dox (*IC_50_* = 38.6 nM). Therefore, this AAP system would be helpful to improve affinity and specificity of antibody or aptamer to their targets in new drug development and existing highly toxic drug targeted delivery systems, especially for therapeutic development for many malignancies [21].

Generally, aptamers are used in combination with antibodies without chemical conjugation. For example, anti-platelet-derived growth factor (PDGF) aptamer E10010 combined with anti-VEGF antibody ranibizumab shows higher therapeutic potency than antibody alone for wet AMD treatment, and have passed phase II clinical evaluation and are waiting for phase III clinical trial now (clinical trial IDs NCT01944839 and NCT01940900) [18,19]. On the other hand, combination therapeutics of anti-PDGF antibody with anti-VEGF aptamer also has promising therapeutic effects.

## 3. Aptamer Selection and Modifications

### 3.1. Systematic Evolution of Ligands by EXponential Enrichment (SELEX)

#### 3.1.1. Conventional SELEX

The method used to develop aptamers is a process called SELEX, which was originally performed and described by Gold and Ellington individually in the 1990s [3,4].

**Figure 2:**
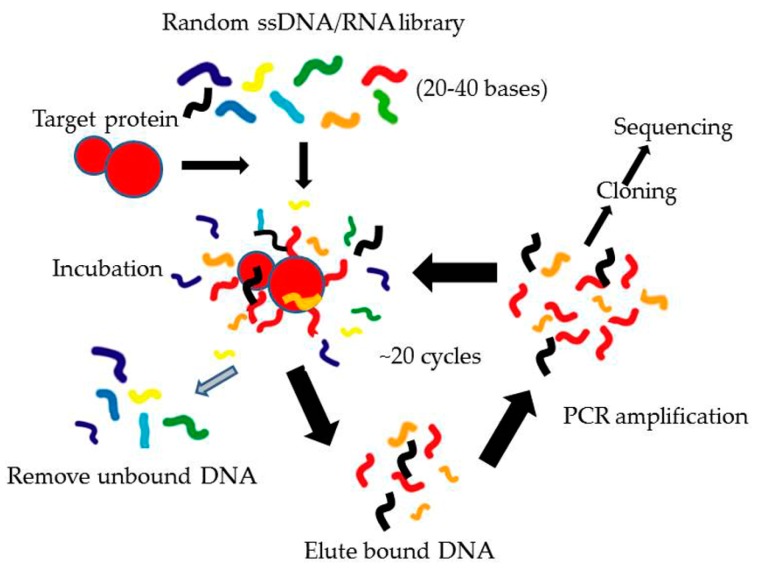
Process of conventional Systematic Evolution of Ligands by EXponential enrichment (SELEX). Different sequences of ssDNA/RNA are shown in different color.

There are several steps in SELEX to find and develop specific aptamers (Figure 2). The first step is synthesis of a screening library, which contains a large number of randomly combinatorial ssDNA and/or ssRNA. All random ssDNA/RNAs have one conserved sequence at each end used for primer binding and amplification and a central random region. The length of the random sequence is normally 20–40 bases so the number of sequences in the whole library would be 10^12^–10^15^, which is enough for library diversity. The second step is to incubate target proteins with the random library under proper conditions. Then through a partition step, the sequences that bind to target proteins are separated from those that do not bind. In the third step, the binding sequences are eluted and amplified using the PCR method (for ssDNA) or RT-PCR (for ssRNA) based on the conserved primer sequences. After these steps, a single cycle of SELEX is completed, which would obtain only a small number of binding sequences. Then in the last step, the selection process is repeated for about 7–20 rounds of incubation, partitioning and amplification, resulting in identification of a small number of binding sequences with high affinity and specificity for further processing and optimization. Generally, the binding sequences are then transformed into bacteria (*E. coli*) for further sequencing as well as characterization. In the post-SELEX process, the specific aptamers can be chemically modified to stabilize and protect them against nucleases *in vivo*. This is the general process of conventional SELEX.

#### 3.1.2. Modified SELEX

As mentioned above, aptamers identified from conventional SELEX process which have high specificity may fail to stimulate or inhibit their targets as expected. The successful rate of effective aptamer identification by conventional SELEX is lower than 30% [21]. Therefore, optimization or variations based on the conventional SELEX may be required in most cases. There are several modifications which are useful and used in research widely (Table 1) [22]. Counter (negative) SELEX is usually performed after positive SELEX to exclude aptamers which bind to negative targets to discriminate highly similar structures [23,24]. Using conventional SELEX in combination with counter SELEX strategy, aptamers that specifically recognized osteoblasts but did not enter hepatocytes and peripheral blood cells were successfully developed for targeted delivery of therapeutic siRNA [24]. This counter SELEX strategy could effectively decrease adverse effects caused by off-target phenomenon *in vivo*. Toggle SELEX is a selection method in which multiple positive targets are used to select aptamers that bind to all targets [25]. For example, drug resistance was frequently found in breast cancer patients when treating with monoclonal antibodies against HER2. Mutation of HER2 on antibody binding sites is one of the major reasons for drug resistance. Aptamers that bind to two different sites on HER2 could still be effective with one of the binding sites mutated (data not shown). Cell SELEX is using live cells to select aptamers for targets which are difficult to be identified or isolated [24,26]. If a genome sequence is used as a library for selection to obtain natural sequences in genomes that can bind to particular targets, it is called genomic SELEX [27,28].

**Figure 3:**
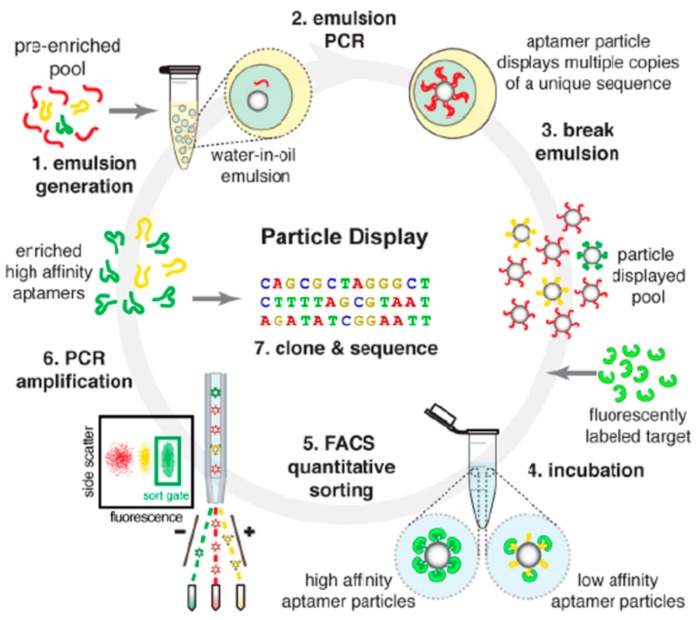
Mechanism of the aptamer particle display system (Reproduced with permission from Reference [29]). Different sequences of ssDNA/RNA are shown in different color.

It is worthwhile to mention that a recently developed aptamer particle display technique can yield aptamers with rather high affinity just in few rounds of SELEX (Figure 3) [29]. In this method, random ssDNA/RNA library is first pre-enriched with several rounds of conventional SELEX and then transformed to particle phase by emulsion PCR with modified primers. Each particle displays multiple copies of one identical sequence on its surface which could be called monoclonal particles. Monoclonal particles are then incubated with fluorescently labeled targets for binding. After removal of non-specific binding targets, fluorescence-activated cell sorting (FACS) could be employed to separate monoclonal particles according to the intensity of fluorescence signal. Using FACS, affinities of over 108 aptamer particles could be measured each time during SELEX which is rather high-throughput. High affinity aptamers are then cloned and sequenced. This technique can dramatically increase the successful rate of aptamer selection, especially for targets which failed to develop aptamer through conventional SELEX [29]. It offers an efficient and economic approach to generate aptamers with high quality, which is helpful for therapeutic aptamer development.

### 3.2. Modifications of Aptamers for Preclinical Studies

**Figure 4:**
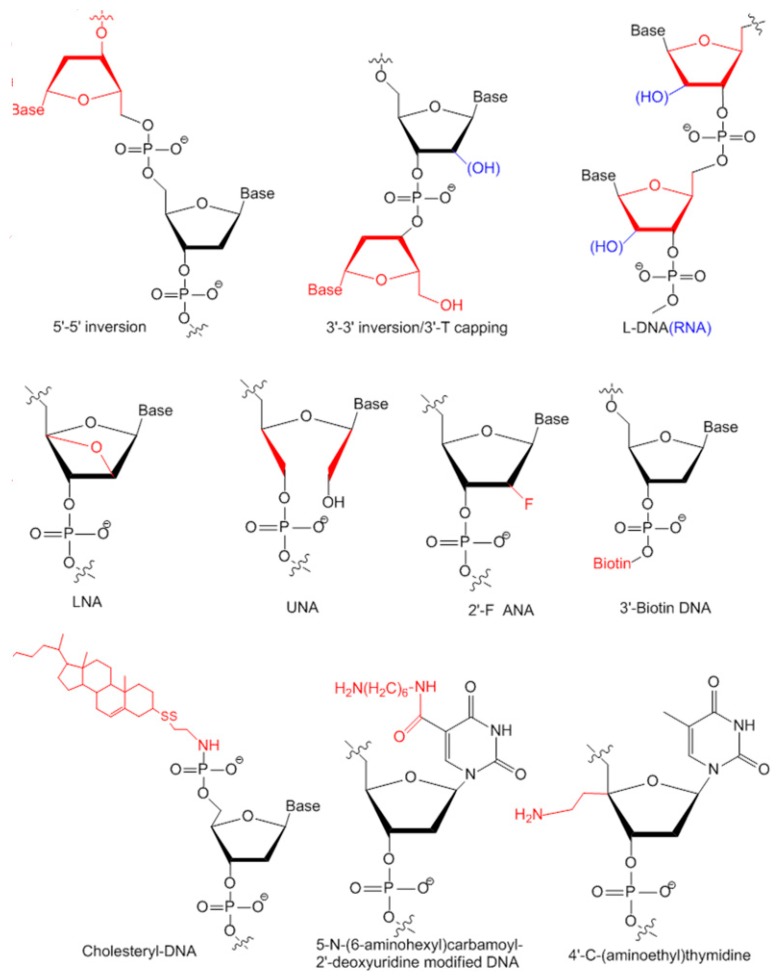
Various chemical modifications to stabilize aptamers (Adapted from Reference [30]). Modification sites are shown in red. The hydroxyl group of RNA is shown in blue to distinguish DNA and RNA.

For therapeutic purposes, as oligonucleotide aptamers could be degraded easily in serum, modifications after SELEX are required for stabilization. Various chemical modifications can significantly improve the stability of aptamers (Figure 4) [30].

#### 3.2.1. Modifications on Linkage

The 15-mer guanine rich (G-rich) thrombin aptamer d(GGTTGGTGTGGTTGG) is a typical and popular model used for developing and illustrating of novel characterization or modification methods for aptamers [31]. It has a G-quadruplex structure formed by two stacking G-tetrads and a central loop TGT which is optimal for stability. It is interesting to find that adding an extra guanine at the 5′ end caused decreased stability while adding an extra guanine to the 3′ end caused increased stability, indicating that the themostability of an aptamer is sequence dependent [32]. It was found that the G-quadruplex is a rather common structure for aptamers, especially for DNA aptamers. DNA aptamers against various targets have a G-quadruplex structure with high sequence identity with high diversity. Another group tried to invert 5′–5′ of a polarity site to form a folded aptamer with a non-common structure d(GGTTGGTGTGGTTGG). This structure has higher stability and affinity to thrombin, although lower inhibiting activity compared to unmodified aptamers [33,34]. Terminal 3′–3′ and 3′–5′ internucleotide linkage was first tried in 1991. A sense deoxyoligonucleotide capping of both ends of the aptamer with inverted thymidine could not only increase stability significantly but also slowed nuclease degradation from 30 min (unmodified) down to 90 min (modified) in snake venom phosphodiesterase digestion [35]. 3′-capping with inverted thymidine modification is a commonly used approach to block 3′-exonuclease degradation by nucleases and prolong an aptamer’s half-life time in serum [23,36]. Most aptamers in clinical trials are modified with this method (Table 2).

Besides inverted thymidine modification, 3′-biotin-streptavidin conjugation is also designed to fight with 3′-exonuclease digestion in serum. It is found that 3′-biotin-streptavidin conjugating aptamers which have bigger size is not only protected from nucleases degradation, but also protected from rapid clearance by circulation system *in vivo* [37]. 3′-biotin modification is also used for aptamers against other targets. Anti-SARS coronavirus helicase aptamer can remain intact for up to 16 h in 10% fetal bovine serum compared to 6 h for unmodified aptamers [23].

Cholesterol can be added to the 5′-end of an aptamer to form a cholesteryl-oligonucleotide (cholODN) and further linked to low density lipoprotein (LDL) to form a compact cholODN-LDL complex. This complex has high stability and is highly resistant to nucleases degradation in serum which has a 10-fold longer half-life than unmodified aptamers [38]. Substitute phosphodiester linkage of DNA with methylphosphonate or phosphorothioate is also commonly used for aptamer stabilization [39,40,41].

#### 3.2.2. Modifications on Sugar Ring or Bases

The natural oligonucleotides are all in d-form. l-form oligonucleotides (Spiegelmer) are chiral inversions of natural d-forms. In identification of l-form aptamers, d-form aptamers are firstly selected against synthesized l-form protein targets from a general single strand random oligonucleotide library. After SELEX, l-form aptamers are synthesized according to the mirror image of corresponding d-form aptamers [43]. l-form aptamers are much more stable than d-form with high resistance to nuclease degradation *in vivo* and do not hybridize to or affect the original nucleic acids in the cells [44,45,46,47]. Clinical evaluated aptamers NOX-A12, NOX-H94 and NOX-E36 are all l-form aptamers.

Locked nucleic acid (LNA) is a modification on a sugar ring with a methylene linkage between 2′-O and 4′-C, which can generate the most stable pairs to dramatically increase the themostability and nucleases resistance of aptamers [48,49]. The LNA/DNA chimera aptamer against HIV-1 trans-activating response target could retain an intact structure without degradation for up to 20 h in serum [48].

Unlocked nucleic acid (UNA) is an opposite modification to LNA. There is a bond between C2′ and C3′ missing in UNA which makes aptamer more flexible. Different from LNA which can stabilize structure, UNA has an uncertain affect to themostability of aptamers. It is found that UNA replacement on a loop region of an anti-thrombin aptamer increased its themostability while replacement on G-tetrads disrupted the structure formation [50]. It is uncertain whether UNA modification has any effect on protecting aptamers from nucleases degradation [50].

Other positions of the sugar ring could be amended for chemical modifications, such as 2′-F [39,51,52], 4′-C-(aminoethyl) thymidine [51], 5-*N*-(6-aminohexyl)carbamoyl-2′-deoxyuridine [39,51] and so on. For more modifications in detail, please read reviews written by Wang *et al.* [30]. More studies are required to characterize and discuss the stabilizing effects of these modifications and design more modification strategies in the future.

## 4. Aptamers for Skeletal Diseases Therapy in Preclinical Studies

The number of bone marrow mesenchymal stem cells (BMSCs) is decreased through aging while increased through adipocyte differentiation. It is found that miR-188 level is much higher in BMSCs in old than in young mice and human. Animals lacking miR-188 can be protected from age-related bone loss and fat accumulation in bone marrow. An aptamer that specifically recognizes BMSCs is developed and conjugated with miR-188 to form a nanocomplex. This targeted delivery nanocomplex could promote bone formation and reduce fat accumulation in bone marrow with high efficacy in aged mice, indicating a potential approach for age-related bone loss therapy [53].

Furthermore, BMSCs are important in bone marrow but there is no specific marker on their surface, making it difficult to be isolated from bone marrow directly. Aptamers with high binding affinity against porcine BMSCs are developed by SELEX. Using the high affinity and specificity aptamers, BMSCs could be fished out from cell solution and bone marrow, which is a novel method for BMSCs isolation and provide a foundation for aptamer applications in tissue engineering and regenerative medicine for skeletal diseases therapy [54].

In another preliminary study, a specific aptamer against human jaw periosteal cells (JPCs) is developed for tissue engineering in oral and maxillofacial surgery. This aptamer has high affinity to human osteogenically induced JPCs and BMSCs from bone marrow while it does not bind to any other cell lines or undifferentiated JPCs or JPCs induced from other sources. It can be used to purify osteogenic progenitor cells from undifferentiated JPCs or stem cells of other sources. The mineralization capacity is higher in the aptamer positive fraction, which is a promising technique for tissue engineering [55].

There are other aptamers or other aptamer-ligand complexes developed and studied in preclinical research at the moment. However, there is no aptamer evaluated in clinical trials. More efforts and studies are needed for this therapeutic field in the future.

Adult mesenchymal stem cells (aMSCs) are stem cells.

## 5. Aptamers in On-Going or Completed Clinical Trials for Therapeutics

At the moment, there are 11 aptamers evaluating in clinical trials for the treatment of macular degeneration, cancer, inflammation and coagulation, and one of them has been approved by FDA for the treatment of AMD. In this section, we will review the research and clinical evaluation progress of these aptamers.

### 5.1. Aptamers against Macular Degeneration

Wet (neovascular) and dry (atrophic) AMD are two major causes of vision loss in the elderly due to retinal damage, and affect around eight million people in America. There are three aptamers being evaluated in clinical trials now for wet or dry AMD therapy.

#### 5.1.1. Pegaptanib

Pegaptanib (Macugen; Pfizer and Eyetech, New York, NY, USA), a 27-mer RNA aptamer specifically binds to and inhibits VEGF against AMD, is the only aptamer approved by the FDA for disease treatment on the market [56]. Pegaptanib is selected directly against VEGF_165_, the VEGF isoform primarily responsible for pathological ocular neovasculariztion and vascular permeability [8]. After *in vitro* selection and characterization, the aptamer which inhibits VEGF_165_ with high affinity and efficacy is chosen and modified with 2′-fluoro pyrimidines and 2′-*O*-methyl purines and further capped with 3′–3′-linked deoxythymidine to avoid nuclease degradation and increase stability. In a preclinical animal study, a 40 kDa polyethylene glycol (PEG) is conjugated to the 5′- end of the RNA aptamer to increase half life of the aptamer for better bioavailability. After around 10 years preclinical studies to optimize and evaluate its therapeutic potency, pegaptanib which shows high efficacy in inhibiting VEGF in different models was approved by the FDA for the treatment of AMD in 2004 with dosage of 0.3 mg per eye every 6 weeks administered intravitreally [56]. This is extraordinary progress as it is the first aptamer approved for use in human and it opens a wide window for therapeutic aptamers in disease treatment.

However, pegaptanib failed to compete with anti-VEGF monoclonal antibody ranibizumab (Lucentis; Genentech, South San Francisco, California, USA) as it only inhibits VEGF_165_ isoforms, while antibodies inhibit all isoforms of VEGF [57], which has negative affects on the pharmacy investment to therapeutic aptamers in these years. Fortunately, it was found recently that blocking all activity of VEGF may cause high risk of hypertension and other adverse effects [58]. Therefore, aptamer pegaptanib is better than antibody ranibizumab in long term maintenance therapy, especially in patients with systemic comorbidities [18,59].

#### 5.1.2. ARC1905

ARC1905 (Ophthotech Corp, New York, NY, USA) is a 39 bases RNA aptamer specifically against complement component 5 (C5) for the treatment of both wet and dry AMDs [60]. C5 is a downstream pro-inflammatory protein in the complement system associated with AMD pathogenesis. Inhibition of C5 can prevent the key terminal fragments formation which is critical for tissue pathology [61]. A PEG is also conjugated to the 3′- end with an inverted thymidine. A phase I clinical trial using ARC1905 in combination with ranibizumab for the treatment of wet AMD (NCT00709527) was finished in 2011 and a phase I clinical for dry AMD treatment (NCT00950638) has recently been completed (data not shown).

#### 5.1.3. E10030

It may not be sufficient to inhibit VEGF only for wet AMD treatment to prevent angiogenesis due to the limit of new vessels regression associated with vision loss. PDGF plays an important role in pericyte recruitment and maturation and new vessels may resist to anti-VEGF drugs due to the role of PDGF [19,62]. Combination treatment using pegaptanib and anti-PDGF antibody can not only prevent new vessel formation but also promote vessel regression [19]. On the other hand, combination treatment using anti-PDGF aptamer and anti-VEGF antibody also has promising therapeutic effect. A 29 bases RNA aptamer E10030 (Fovista; Ophthotech Corp) targeting PDGF is developed and modified with 2′-fluoro pyrimidine and 2′-O-methyl purines. In preclinical studies, PEG-conjugated E10030 was able to facilitate neovascular regression when combined with anti-VEGF agents [19]. In clinical trials, patients treated with E10030 in combination with anti-VEGF antibody raninizumab (administered once a month) showed significant neovascular regression and 59% of them have increased visual acuity after three months of treatment without any side effects observed. In phase II clinical trials of this combination therapy, patients treated with combination therapy gained 62% higher vision than the patients treated with anti-VEGF antibody ranizumab only. The combination therapy is waiting for phase III clinical evaluation for wet AMD treatment at the moment (clinical trial IDs NCT01944839 and NCT01940900). The promising results suggest that combination therapy targeting two different antigens at the same time using aptamer-aptamer combination or aptamer-antibody combination could be a new therapeutic direction in future study.

### 5.2. Aptamers against Cancer

Cancer treatment requires more effective and precise therapies, especially specific therapies, for discriminating normal cells and tumor cells to avoid toxicity. The highly disorganized vessel architecture inside the tumors and the surrounding extracellular matrixes as well as stromal cells are both barriers for drug delivery [63]. Monoclonal antibodies can recognize targeting tumor cells with high specificity but are difficult to penetrate into the deep sites of tumor cells due to the large size (around 150 kDa). Aptamers with much smaller size (around 30 kDa) are able to cross the barriers and penetrate into tumor cells and therefore are ideal therapeutic reagents for cancer. Generally, aptamers could diffuse into tumors in 10 min after injection. For example, a fluorescent aptamer against extracellular matrix protein tenascin-C was able to diffuse into tumors rapidly with perivascular fluorescence signal detected in tumor only 10 min after intravenous injection [64]. Furthermore, another group has compared the tumor penetration times between aptamer and antibody, and found that they were detected in tumors after 10 min and 3 h after intravenous injection, respectively [65]. Immuno-therapy of cancer by aptamers is a recent novel research focus. There are two individual aptamers for cancer therapy now being evaluated in clinical trials.

#### 5.2.1. AS1411

AS1411 (Antisoma, London, UK) is a guanine-rich aptamer with G-quadruplex structure identified from a guanine-rich ssRNA library by antiproliferation selection [66]. It has a G-quadruplex structure which is highly stable and resistant to nuclease degradation. It can penetrate into tumor cells easily. AS1411 binds to the external domain of nucleolin, which is a protein over-expressed on the surface of cancer cells and responsible for survival, growth, and proliferation of cells [66]. AS411 can inhibit over 80 types of cancer cells in *in vitro* studies and it is the first oligonucleotide aptamer approved for clinical trial for human cancer therapy. In preclinical studies, AS1411 has inhibition efficacy in multiple cancer models including non-small cell lung, renal cells and breast cancers. In phase I clinical trial (NCT00881244), AS1411 is well tolerated by patients with advanced cancers and has no side effects. AS1411 shows promising therapeutic efficacy especially for patients with renal cell carcinoma after six months of therapy. Phase II clinical trial shows therapeutic efficacy to acute myeloid leukemia patients without toxicity and adverse effects. However, a following phase II evaluation for renal cell carcinoma (clinical trial ID NCT00740441) found AS1411 only has therapeutic effect in 2.9% of patients, and shows minimal activity in unselected patients with metastatic renal cell carcinoma [67], indicating more research is required to optimize the therapeutic potency of AS1411 in the future.

#### 5.2.2. NOX-A12

NOX-A12 (Olaptesed pegol; Noxxon, Berlin, Germany) is a 45-mer l-RNA aptamer developed for use in autologous hematopoietic stem cell transplants [66]. NOX-A12 targets to stroma cell-derived factor-1 The chemokine (C–X–C motif) ligand 12 (CXCL-12), which plays important roles in stem cell migration towards the bone marrow and controls tumor growth, metastasis and vasculogenesis. Binding to CXCL-12 can block its receptor binding and prevent CXCL-12 tissue gradients and decrease the possibility of tumor metastasis and drug resistance caused by cancer cell homing [68]. As l-form aptamers can not be recognized by nucleases, it does not require any chemical modifications for *in vivo* studies. It showed efficacy against non- Hodgkin’s lymphoma and myelomas in preclinical studies. Phase I clinical trial for safety and tolerability evaluation confirms NOX-A12 can be well tolerated without serious adverse effects. NOX-A12 has a 37 h long half-life and patients with NOX-A12 treatment are more susceptible to chemotherapy due to its particular blockage of stromal cell-derived factor-1 (SDF-1), making it a promising and successful drug in cancer therapy [69]. Two phase II clinical trials are in progress, one for treatment of Chromin Lymphocytic Leukemia (clinical trial ID NCT01486797) and the other one for evaluating and comparing the therapeutic efficacy of NOX-A12 alone and combination therapy with chemotherapy for patients with multiple myeloma (clinical trial ID NCT01521533).

### 5.3. Aptamers against Coagulation

More and more aptamers for coagulation therapy have been developed independently by different research groups. Till now, there are four aptamers being evaluated in different stages of clinical trials.

#### 5.3.1. REG1

REG1 (Regado Biosciences, Basking Ridge, NJ, USA) is an aptamer system consisting of a 37-mer RNA aptamer RB006 (Pegnivacogin) and a 17-mer antidote RB007 with sequence complementary to RB006 [70]. RB006 is an antagonist of factor IXa for preventing the downstream conversion of factor X and avoidance of clotting. RB007 is the antidote of RB006, which can specifically reverse the inhibition function of RB006 to control sheath removal time after percutaneous coronary intervention. Phase I clinical trials have shown REG1 is well tolerated in patients and no significant adverse effects or major bleeding are found [71,72,73]. More importantly, therapeutic effects of REG1 can be controlled by dosage of RB007 and it is less toxic than heparin/protamine, which is also an anticoagulation/antidote pair currently available [74]. Phase II clinical trials for using REG1 in percutaneous coronary intervention for patients with coronary artery have just been completed (clinical trial ID NCT00715455). However, a later randomized clinical trial had to be terminated before it finished as patients treated with REG1 showed severe allergic reactions and major bleeding with no significant efficacy after percutaneous coronary intervention [75]. Therefore, more studies are required to evaluate the safety and efficacy of the REG1 system.

#### 5.3.2. ARC1779

ARC1779 (Archemix Corp, Cambridge, MA, USA) is a 39-mer PEGylated DNA aptamer that binds to the A1 domain of von Willebrand factor (vWF) [76], which is a key factor in the coagulation cascade related to platelet recruitment, to block interaction between A1 domain and platelet receptor glycoprotein 1B [12,77]. Therefore, it is a potential therapeutic target for treatment of vWF-related platelet disorders, von Willebrand disease as well as acute coronary syndromes [78]. A phase II pilot study showed that ARC1779 can inhibit platelet depletion induced by a vWF agonist desmopressin in patients [79]. Phase II clinical trial for VWF 2D treatment is still on-going.

#### 5.3.3. NU172

Aptamer NU172 (ARCA Biopharma, London, UK) is an unmodified DNA aptamer for short-term anticoagulation and is distinct from the long-term anticoagulation aptamer REG1 and ARC1779. In preclinical studies, NU172 showed efficacy to prolong clotting time and the anticoagulation effect would be stopped rapidly due to nuclease degradation. A phase II clinical trial for evaluating the therapeutic effects in coronary artery bypass graft surgery is in progress (clinical trial ID NCT00808964).

#### 5.3.4. BAX499

The above three anticoagulation aptamers all target to proteins in the intrinsic coagulation pathway, while BAX499 (Baxter, Deerfield, IL, USA) targets to the negative regulator of factor VIIa in the extrinsic tissue factor pathway. In a preclinical hemophilia pathology mimicking monkey model, BAX499 is able to recover the clotting caused by anti-factor VIII antibody [80]. The phase I clinical trial was started from 2010 (clinical trial ID NCT01191372) and the evaluation results have not been published yet.

### 5.4. Aptamers against Inflammation

Two anti-inflammation aptamers in clinical trials are both l-form aptamers from Noxxon.

#### 5.4.1. NOX-H94

Hepcidin is over-expressed in patients with chronic inflammation induced by cancer or dialysis, which may lead to anemia due to the hepcidin-induced ferroportin degradation [81]. NOX-H94 (Lexaptepid pegol; Noxxon) targets to hepcidin, a peptide hormone regulator for iron homeostasis with the role to inhibit the interaction between hepcidin and ferroportin and reduce the anemia symptom in patients [81,82]. In a preclinical study, cynomolgus monkey could be prevented from interleukin-6 induced iron concentration decrease in serum when treated with NOX-H94. Phase IIa clinical trial for anemia therapeutic evaluation for patients with cancers have been completed. Clinical trials for treatment of erythropoiesis agent-induced anemia in patients with dialysis are still in progress (clinical trial ID NCT02079896).

#### 5.4.2. NOX-E36

NOX-E36 is another l-form anti-inflammation aptamer from Noxxon [83]. The target of NOX-E36 is chemokine ligand 2 (also called monocyte chemoattractant protein 1) which can mediate inflammation by recruiting leukocytes from intravascular to extravascular environments. Upon binding, NOX-E36 can inhibit chemokine ligands 2-induced inflammation to reduce the recruitment of leukocytes, which is quite effective for anti-inflammation treatment for lupus nephritis in mouse models. It is also useful for preventing type-2 diabetic glomerulosclerosis in mice [84]. Phase II clinical trials for treatment of type-2 diabetes are in progress (clinical trial IDs NCT01085292 and NCT01547897).

## 6. Conclusions

Oligonucleotide aptamers are more and more popular in recent years, especially from 2005 after the first aptamer Pegaptanib was approved for wet AMD therapy by FDA. There are over 900 aptamers developed by SELEX for a broad spectrum of both diagnostic and therapeutic applications. You can find over 5000 reports on aptamer research in PubMed, and this number currently generally increases by two to five every day. There are 11 aptamers in clinical trials, which have advantages especially for safety issues and have significant improvements in efficacy for therapeutic application. In addition to these aptamers, there are nearly 100 aptamers waiting for approval for evaluation in clinical trials.

With promising advantages compared to monoclonal antibodies, oligonucleotide aptamers may become the predominant agent for therapeutic application. With monoclonal antibody technology commonly developed, and used in therapeutic application, the targets of antibodies are well characterized [85] (Table 3). Therefore in the future, based on the promising therapeutic effects of inhibiting particular targets with monoclonal antibodies, oligonucleotide aptamers can be identified against the same targets to develop the second generation of therapy. This would save considerable research effort and allow more rapid progress in developing more economic and efficient therapeutic approaches for various diseases with oligonucleotide aptamers.

## Figures and Tables

**Table 1:** List of modified Systematic Evolution of Ligands by EXponential Enrichment (SELEX) methods which are commonly used.

Name	Property	Advantages
Counter SELEX	Introduce negative selection to exclude aptamers bind to negative target	Could discriminate highly similar structure to increase specificity
Toggle SELEX	Multiple positive selection targets	Could select aptamers bind to multiple targets
Capillary electrophoresis-SELEX	Separate aptamer–target complexes from free aptamers according to their electrophoretic mobility with capillary electrophoresis	Could effectively identify high affinity aptamers in four rounds
Cell SELEX	Select against whole cells	No prior target knowledge required
*In vivo* SELEX	generate aptamers in living organisms	No prior target knowledge required Suitable for cancer therapy as tumors have high varieties and *in vitro* selection may not work
*In silico* SELEX	Employ computational docking	Could be used to predict aptamer affinity, specificity, 3D structure and aptamer-target interaction by computer prior to experimental characterization
SELEX with high-throughput sequencing	Could use high-through sequencing after each round of selection.	Could be used for selection of a large number of aptamers. Could identify aptamers in two to three rounds of SELEX and could perform comprehensive characterization of identified aptamers.

**Table 2:** Progress of aptamers for diseases therapy in on-going or completed clinical trials [42].

Therapeutic Purpose	Name	Target	Form	Modification	Status	Section
Macular degeneration	Pegaptanib	Vascular endothelial growth factor (VEGF)	RNA	2′-fluoro pyrimidines, 2′-*O*-methyl purines, 3′-inverted dT, PEGylated	Approved for age-related macular degeneration (wet AMD)	5.1.1
	ARC1905	Complement component 5	RNA	3′-inverted dT, PEGylated	Phase I completed	5.1.2
	E10030	Platelet-derived growth factor (PDGF)	DNA	2′-fluoro pyrimidines, 2′-O-methyl purines 3′-inverted dT	Phase III await	5.1.3
Cancer	AS1411	Nucleolin	RNA	G-rich, PEGylated	Phase II on-going	5.2.1
	NOX-A12	The chemokine (C–X–C motif) ligand 12 (CXCL-12)	l-RNA	l-form, PEGylated	Phase II on-going	5.2.2
Coagulation	REG1	Coagulation factor IXa	RNA	3′-inverted dT, PEGylated	Phase III await	5.3.1
	ARC1779	von Willebrand factor (vWF) A1 domain	DNA	3′-inverted dT, PEGylated	Phase II on-going	5.3.2
	NU172	Thrombin	DNA	Unmodified DNA	Phase II on-going	5.3.3
	BAX499	Tissue factor pathway	RNA	3′-inverted dT, PEGylated	Phase I on-going	5.3.4
Inflammation	NOX-H94	Hepcidlin	l-RNA	l-form, PEGylated	Phase II on-going	5.4.1
	NOX-E36	The chemokine (C–C motif) ligand 2 (CCL2)	l-RNA	l-form, PEGylated	Phase II on-going	5.4.2

**Table 3:** Monoclonal antibodies approved by FDA for therapeutic use.

Antibody	Trade Name	Target	Approved Indication
Muromomab	Orthoclone	CD3	Allograft rejection in allogeneic renal transplantation
Abciximab	ReoPro	Glycoprotein IIb/IIIa	Percutaneous coronary intervention
Rituximab	Rituxan	CD20	RA, Wegner granulomatosis, microscopic polyangiitis
Daclizumab	Zenapax	CD25 (II2r)	Allograft rejection
Basiliximab	Simulect	CD25 (II2r)	Allograft rejection
Palivizumab	Synagis	Protein F	Respiratory syncytial virus (RSV inhibitor) in children
Infliximab	Remicade	TNFα	Crohn’s disease and rheumatoid arthritis
Trastuzumab	Herceptin	HER2/Neu	Metastatic breast cancer
Etanercept	Enbrel	TNFα and β	Autoimmune diseases such as ankylosing spondylitis
Gemtuzumab	Mylotarg	CD33	CD33-positive acute myeloid leukemia
Alemtuzumab	Mabcampath	CD52	B-cell chronic lymphocytic leukemia
Ibritomomab	Zevalin ^90^Y	CD20	B-cell non-Hodgkin’s lymphoma
Adalimumab	Trudexa	TNFα	Crohn’s disease and rheumatoid arthritis
Alefacept	Amevive	CD2	Chronic plaque psoriasis
Omalizumab	Xolair	IgE	asthema
Tositumomab	Bexxar	CD20	CD20-positive B-cell non-Hodgkin’s lymphoma
Efalizumab	Raptiva	CD11a	Moderate to severe plaque psoriasis
Cetuximab	Erbitus	EGFR	Metastatic colorectal and head and neck carcinoma
Bevacizumab	Avastin	VEGF-A	Metastatic colorectal and non-small cell lung carcinoma
Natalizumab	Tysabri	Integrin-α4	Multiple sclerosis
Ranibizumab	Lucentis	VEGF-A	Wet type age-related macular degeneration
Panitumumab	Vectibid	EGFR	Metastatic colorectal carcinoma
Eculizumab	Soliris	C5	Paroxysmal nocturnal haemoglobinuria
Certolizumab	Cimzia	TNFα	Crohn’s disease
Daratumumab	Darzalex	CD38	Multiple myeloma
Elotuzumab	EMPLICITI	CS1	In combination with lenalidomide and dexamethasone for Multiple myeloma
Mepolizumab	Nucala	IL-5	Asthma
Denosumab	Prolia/Xgeva	Nuclear factor kappa B ligand	Bone matastases, osteoporosis, giant cell tumor of bone
Secukinumab	Cosentyx	IL-17	Psoriasis
Sirukumab	(CNTO 136)	IL-6	Rheumatoid arthritis (soon)

## Abbreviations

The following abbreviations are used in this manuscript:

SELEX: Systematic Evolution of Ligands by EXponential enrichment
AMD: Age-related Macular Degeneration
AAPs: Antibody-aptamer pincers
FACS: Fluorescence-Activated Cell Sorting
cholODN: cholesteryl-oligonucleotide
LDL: low density lipoprotein
VEGF: vascular endothelial growth factor
PDGF: Platelet-Derived Growth Factor
vWF: von Willebrand factor
CD: Cluster of Differentiation
IL: InterLeukin
EGFR: Epidermal Growth Factor Receptor
sulfo-SMCC: sulfosuccinimidyl 4-(*N*-maleimidomethyl) cyclohexane-1-carboxylate
SATA: *N*-succinimidyl-S-acetylthioacetate
CXCL-12: The chemokine (C–X–C motif) ligand 12
